# Primary Extramedullary Acute Promyelocytic Leukemia Presenting as Cauda Equina Syndrome: A Rare Case Report and Diagnostic Challenge

**DOI:** 10.1002/ccr3.72767

**Published:** 2026-05-29

**Authors:** Amir Ali, Marc Ringor, Arbab Khalid, Diana Garcia, Ravishankar Konchada, Ramalingam Ratnasabapathy, Yuna Chae, Arman Melikyan, George Yaghmour

**Affiliations:** ^1^ University of Southern California Norris Comprehensive Cancer Center Los Angeles California USA; ^2^ Department of Internal Medicine Kirk Kerkorian School of Medicine at UNLV Las Vegas Nevada USA; ^3^ Private Practice, Family Medicine Physician Santa Maria California USA; ^4^ Radiology St. Rose Dominican Hospitals Las Vegas Nevada USA; ^5^ Medical Oncologist and Hematologist, Horizon Ridge Henderson Henderson Nevada USA; ^6^ USC Mann School of Pharmacy Los Angeles California USA; ^7^ Internal Medicine, Jane Anne Nohl Division of Hematology and Center for the Study of Blood Diseases University of Southern California Norris Comprehensive Cancer Center Los Angeles California USA

**Keywords:** acute medicine, hematology, oncology, pathology and laboratory medicine

## Abstract

APL presenting as an extramedullary mass is exceedingly rare. Here, we describe extramedullary APL causing cauda equina syndrome, underscoring the diagnostic challenges associated with an atypical presentation, highlighting the complexity of distinguishing extramedullary APL from other conditions, and discussing the stepwise approach to diagnosis.

## 1 Introduction

Cauda equina syndrome (CES) is a neurological emergency that occurs due to compression of the cauda equina nerve roots, often caused by herniated discs, trauma, infections, or neoplastic processes [1]. Hematologic malignancies rarely present as spinal cord compression, and APL has been infrequently reported in this context.

Acute Promyelocytic Leukemia (APL) is a distinct subtype of acute myeloid leukemia (AML) characterized by the presence of the PML‐RARA fusion gene, which results from the t(15;17) translocation. While APL typically presents with hematologic abnormalities and bone marrow involvement, extramedullary manifestations are rare [2]. Even more uncommon is the presentation of APL as an isolated extramedullary mass without bone marrow involvement.

Here, we describe a rare case of APL manifesting primarily as an extramedullary spinal mass, leading to CES. Unlike typical cases of extramedullary APL, which often arise following chemotherapy for AML or other malignancies, this case was unique in its presentation as an initial, isolated disease manifestation. This report highlights the diagnostic challenges associated with extramedullary APL, the importance of differentiating it from other spinal lesions, and the key histopathological features essential for diagnosis. Additionally, we review therapeutic considerations for managing both CES and extramedullary APL, providing insight into this rare and atypical presentation.

## 2 Case History

Written informed consent was obtained from the patient. A 44‐year‐old gentleman with chronic low back pain presented to the hospital complaining of worsening left lower quadrant abdominal pain radiating to his lower back, as well as intermittent weakness of the lower extremities.

Prior to his presentation, the patient experienced intermittent back pain for a year and a half, as well as 70 pounds of weight loss for two and a half years that he attributed to a healthy diet. The patient also noted smoking a quarter pack of cigarettes daily for several years. On presentation, the patient endorsed several weeks of worsening pain that was exacerbated by ambulation but denied headaches, visual disturbances, saddle anesthesia, or changes in bladder and bowel function. Vital signs were unremarkable, while examination was significant for a palpable mass located left of the abdominal midline measuring approximately 4 cm by 5 cm, along with paraspinal lumbar muscle tenderness. Laboratory results were notable for a reduced white blood cell count of 3.2 K/μL with a lymphocyte percentage of 19.7%.

**FIGURE 1:**
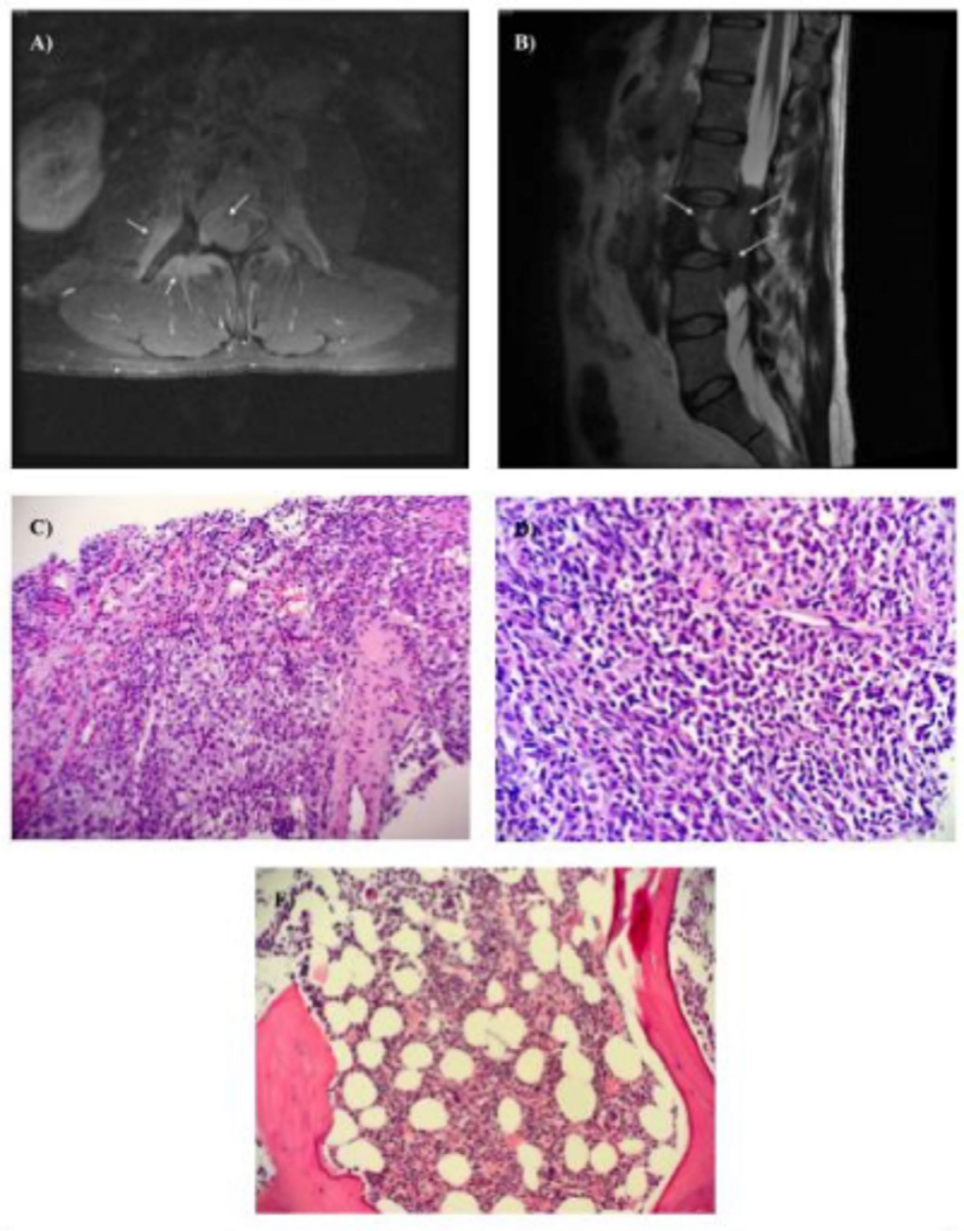
Lumbar Imaging Studies and Histopathology of the Lumbar Mass and Bone Marrow An initial axial T‐2 weighted MRI image of the lumbar spine performed with the administration of intravenous contrast material (Panel A), shows a bony and soft tissue mass centered around the L3 vertebral body. The mass displaces the nerve roots to its left, with anterior displacement or invasion of the left psoas muscle. It also extends right and posteriorly into the spinal canal, with invasion of a small portion of the paraspinous musculature. Retroperitoneal adenopathy is present. A sagittal T2‐weighted image (Panel B) shows an extradural lesion invading through the meninges and into the thecal sac. Intracanalicular stenosis is present with complete effacement of the CSF at the level of L3. H&E staining of a sample collected from the L3 paraspinal lumbar mass (Panel C) is consistent with the presence of a myeloid sarcoma. Magnification of the sample (Panel D) suggests the presence of malignant myelogenous cells. An H&E stain of bone marrow aspirate collected from the left iliac bone (Panel E) shows the absence of leukemic blasts.

A computed tomography (CT) scan of the abdomen and pelvis without contrast revealed a bone lesion located in the L3 vertebra, a spinal canal mass from L2 to L4, and the involvement of several iliac lymph nodes in the retroperitoneum consistent with lymphoma. 1 day later, CT scans of the head with and without contrast, along with a CT scan of the chest with contrast, showed no evidence of metastatic disease. Magnetic resonance imaging (MRI) of the lower lumbar spine with and without contrast showed an extensive spinal lesion centered at the L3 vertebra, suggesting a lymphoid mass measuring 6 cm by 1.1 cm by 1.3 cm (Figure 1A,B). Discrete intracanalicular stenosis with local invasion of the meninges and intrathecal space, retroperitoneal adenopathy, and invasion and displacement of the local vasculature and musculature were discovered.

2 days after his presentation to the emergency department, the patient was formally admitted to the hospital and given dexamethasone to manage his back pain and lower extremity weakness associated with CES. While symptomatic treatment of CES with high‐dose corticosteroids may give temporary pain relief and improve neurologic functioning by reducing edema and/or cord compression, definitive treatment focuses on identifying the underlying cause, including lumbar disc herniation, spinal stenosis, tumor mass effect, infection, or bony ingress [1]. The presence of a paraspinal mass on diagnostic imaging and pathology consistent with myeloid sarcoma suggested the patient's CES was due to tumor mass effect. Although dexamethasone provided significant relief, he later complained of right lower extremity numbness and weakness consistent with lumbar radiculopathy, which was mildly resolved after gabapentin administration. A neurosurgery consultation was obtained, with recommendations for conservative management. On day 2 of hospitalization, a CT‐guided core needle biopsy of the lumbar mass, along with a CT‐guided bone marrow biopsy of the left iliac bone, were performed. Histopathology results of the biopsy were consistent with a myeloid sarcoma (Figure 1C,D) and showed the absence of leukemic blasts (Figure 1E) [2]. On day 3 of hospitalization, laboratory studies were significant for a segmented neutrophil count of 88.9%, a lymphocyte count of 8.2%, and a lactate dehydrogenase of 256 U/L.

On day 7 of hospitalization, an oncology consultation was obtained. Additional samples of the paraspinal mass were sent to a third‐party hematopathologist for further testing, including chromosomal analysis and next‐generation sequencing for AML, to find causal mutations and determine a prognosis. A complete blood count was notable for a white blood cell count of 12.3 × 10[3]/L, a segmented neutrophil count of 86%, a lymphocyte percentage of 5%, and a metamyelocyte percentage of 2%. Additional laboratory findings included a total serum protein of 6.0 g/dL, a serum albumin of 3.0 g/dL, and an alanine transaminase of 56 U/L. On day 10 of hospitalization, a lumbar puncture with cerebrospinal fluid (CSF) analysis showed the presence of leukemic cells, but no concern for acute infection. Other laboratory results, including urinalysis and blood cultures, were unremarkable.

The patient was transferred to the oncology floor and intrathecal administration of methotrexate was recommended due to the presence of leukemic blast in the CSF [3, 4, 5, 6]. On day 8 of hospitalization, hematopathology reported morphologic and immunophenotypic findings from the paraspinal mass biopsy, supporting a diagnosis of myeloid neoplasm, or chloroma [7]. The patient was immediately started on a FLAG‐IDA (fludarabine, cytarabine, filgrastim, and idarubicin) and venetoclax induction chemotherapy regimen for 1 month, as extramedullary presentation put them at a high risk for relapse [8]. Treatment consisted of 400 mg of oral venetoclax daily for 14 days, along with daily 300 mcg injections of filgrastim for the first 7 days [9]. Beginning on day 2 of treatment, the patient received 30 mg/m^2^ of fludarabine and 1.5 g/m^2^ of high‐dose cytarabine intravenously for 4 days. From day 4 to 6 of treatment, 8 mg/m^2^ of intravenous idarubicin was administered concurrently with fludarabine and cytarabine. Allopurinol was given as prophylaxis for tumor lysis syndrome [10, 11, 12]. Shortly after beginning chemotherapy, the patient developed severe pancytopenia. A consultation from infectious diseases recommended starting leukopenic precautions and empiric antimicrobial administration of levofloxacin, posaconazole, and acyclovir. A regimen of filgrastim was also initiated. The patient continued to experience pancytopenia during treatment, requiring multiple platelet transfusions [12, 13]. The remainder of their hospitalization was unremarkable, with no signs or symptoms of acute hemorrhage or infection and an eventual resolution of the pancytopenia.

On day 27 of hospitalization, a repeat MRI of the lower spine with and without contrast reported a reduction in the size of the chloroma greater than 60%, with improved spinal stenosis. FISH analysis demonstrated the presence of PML‐RARA, as well as a translocation between chromosomes 15 and 17 [t(15;17)], confirming a diagnosis of extramedullary APL. On day 35 of hospitalization, an Ommaya reservoir was placed by interventional radiology shortly before the patient's discharge for further outpatient treatment.

## 3 Differential Diagnosis

The first step in creating a differential diagnosis in this case was to determine if the patient's presentation was related to the abdominal mass found during their physical exam. After initial diagnostic imaging revealed the appearance of a paraspinal mass consistent with lymphoma, its origin and composition were then assessed. Origins to consider included a sarcoma of the lumbar spine with local invasion of anatomical structures, or metastatic invasion of the lumbar spine from a nearby organ. While immediate chemotherapeutic intervention for AML in accordance with current guidelines was initiated upon confirmation of the mass as a myeloid sarcoma, further immunophenotyping and chromosomal analysis were conducted to identify a specific subtype and prognosis [14].

The combination of CES, discovery of a lumbar spinal canal mass on CT imaging and MRI scans, presence of metamyelocytes on laboratory findings, and results from the core needle biopsy of the paraspinal mass confirmed the presence of a myeloid sarcoma located in the lumbar spine. Although the lack of abnormal findings on bone marrow biopsy of the L3 vertebrae reduced the likelihood of extramedullary leukemia, immunophenotypic studies and FISH analysis of the mass confirmed this diagnosis. The significant reduction of the patient's chloroma after induction chemotherapy further endorsed it as an extramedullary leukemia. Altogether, the features of this case suggest that the patient most likely had CES due to the presence of a large chloroma caused by extramedullary APL.

Based on both a clinical diagnosis of CES due to a large paraspinal mass as well as a laboratory diagnosis consistent with extramedullary APL, a final diagnosis was extramedullary APL with spinal myeloid sarcoma (Table 1).

**TABLE 1:** Additional laboratory data.

Variable	Reference range, adults	Initial presentation to hospital	Day 3 of hospitalization	Day 8 of hospitalization	Day 23 of hospitalization	Day 30 of hospitalization
White Blood Count (WBC)	4.0–12.0 K/μL	3.2	6	12.3	3.9	4
Red Blood Count (RBC)	4.0–6.0 M/μL	4.4	3.9	4.53	3.24	3.36
Hemoglobin (Hgb)	12.5–17.5 g/dL	12.5	11.1	12.9	9.9	10.5
Hematocrit (Hct)	35.0%–50.0%	37.1	32.6	37.9	30.7	32.4
Mean Corpuscular Volume (MCV)	81.0–99.0 fL	84.4	83.4	83.8	95	96
MCH	27.0–34.0 pg	28.3	28.5	28.5	31	31
MCHC	32.0–36.0 g/dL	33.6	34.1	34	32.2	32.4
RDW	12.0%–17.0%	14.3	14.3	14.6	20.5	21.7
Plt	150–400 K/μL	236	305	323	145	159
MPV	6.0–11.0 fL	8.4	9.2	8.4	—	—
Man Segs	41.0%–75.0%	69.1	88.9	86	87	88
Man Bands	0%–5.0%	—	—	0	0	0
Man Lymphs	20.0%–45.0%	19.7	8.2	5	5	6
Man Monos	2.0%–15.0%	7.4	2.9	6	5	4
Man Metas	0%	—	—	2	—	—
Man Myelos	0%	—	—	1	—	—
RBC Morph	Normal	—	—	Normal	—	—
Plt Est	Adequate	—	—	Adequate	—	—

## 4 Conclusion and Results

### 4.1 Management of Primary Extramedullary APL

While the patient's initial therapeutic course was in accordance with guidelines for the treatment of extramedullary AML, the confirmation of extramedullary APL complicated treatment. The current literature for management of primary presenting extramedullary APL is limited, as the vast majority of cases involving extramedullary APL occur after chemotherapeutic treatment for AML or other types of cancer. To date, a review of relevant scientific articles includes 5 case reports that note the presence of a paraspinal chloroma [2, 9, 15, 16, 17]. However, the presentation of this case fundamentally differs from other similar cases due to a lack of leukemic blasts on bone marrow biopsy of nearby vertebrae. Subsequent rounds of diagnostic imaging throughout the body did not detect additional bone lesions. As such, this is the first reported case of a primary presenting extramedullary APL without bone marrow involvement, though the underlying mechanism explaining this occurrence remains unclear. The addition of all‐trans retinoic acid (ATRA) and arsenic trioxide (ATO) after the completion of their first cycle of chemotherapy was discussed with the patient. Due to the increased potential for serious side effects associated with chemotherapy and ATRA, including increased risk of bleeding, ATRA syndrome, and sepsis, the patient was counseled on early symptom recognition and intervention [18, 19, 20]. Notably, ATRA syndrome in patients treated for APL is low, with a prior study suggesting that the early initiation of chemotherapy can reduce its incidence in newly diagnosed APL with leukopenia [21].

### 4.2 Follow‐Up

**FIGURE 2:**
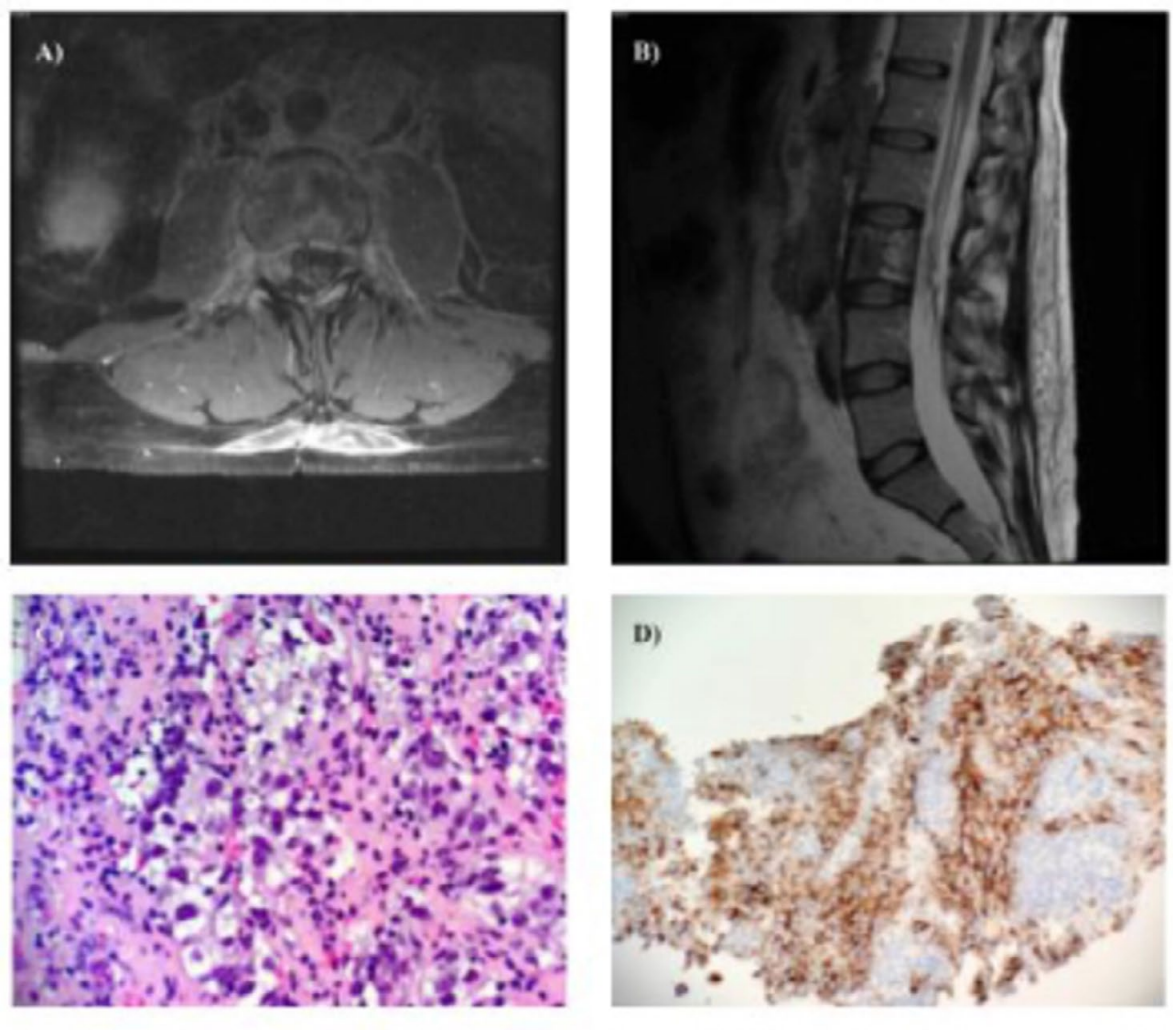
Lumbar Imaging Studies (Post‐discharge Day 37) An axial T‐2 weighted MRI image of the lumbar spine performed after induction chemotherapy and subsequent ATRA‐ATO therapy (Panel A), shows remission of the mass located around the L3 vertebral body, while persistent retroperitoneal lymphadenopathy remained. A sagittal T‐2 weighted image (Panel B) also shows remission of the L3 mass, along with a resolution of the intracanalicular stenosis and restored CSF flow. H&E staining of one of the affected retroperitoneal lymph nodes (Panel C) indicated the presence of a germ cell tumor consistent with a seminoma. Placental alkaline phosphatase (PLAP) immunochemistry of the lymph node biopsy (Panel D) confirms the presence of a seminoma.

The patient was closely monitored after initial hospitalization, continuing his chemotherapy regimen outpatient with the addition of ATRA and daily ATO after its completion. The patient's clinical picture subsequently improved, with decreased lower back pain, improved ambulation, a reduction in the size of the myeloid sarcoma, as well as improved intracanalicular stenosis at the level of L2–L4 on MRI imaging. Over 1 month after discharge from the hospital, the patient presented to their outpatient oncology clinic with a fever of 102°F and cytopenia. The patient returned to the emergency department due to concern for ATRA syndrome versus sepsis [18, 20]. Repeat MRI imaging demonstrated significant remission of the myeloid sarcoma (Figure 2A,B). However, persistent retroperitoneal lymphadenopathy remained when compared to imaging studies from his previous hospitalization.

Histopathology and immunohistochemistry of biopsy samples from one of the affected lymph nodes indicated the presence of a germ cell tumor (GCT) consistent with a seminoma (Figure 2C,D). Since retroperitoneal lymphadenopathy was detected on diagnostic imaging during their initial hospitalization, there may be a relationship between the presence of a GCT and the patient's extramedullary APL, and it may have been present on initial hospitalization. Moreover, a previous study of 15 individuals with non‐seminomatous GCTs and co‐incident hematologic malignancies, including AML, found that a number of mutational similarities between both types of cancer suggest the existence of a progenitor cell lineage with the capacity to differentiate into both malignancies [22]. However, the basis for the specific association between non‐seminomatous GCTs and hematologic malignancies remains unclear. Furthermore, it is uncertain whether one malignancy preceded the other, or if both arose simultaneously.

The patient was placed under hospital observation, and an additional consultation from infectious diseases was obtained. The patient received meropenem and vancomycin, along with continued consolidation therapy including ATRA, ATO, intrathecal methotrexate and cytarabine [3, 4, 5, 6]. The patient was subsequently afebrile with laboratory studies significant for neutropenia but otherwise unremarkable. Given their high‐risk features, the patient will be evaluated for bone marrow transplant after completion of their arsenic therapy [16, 18, 23].

## 5 Discussion

### 5.1 Pathological Discussion

Early laboratory studies during hospitalization revealed an elevated percentage of metamyelocytes. Common causes of this include leukemia, infection, medications associated with treatment for AML or its subtypes, asplenia/hyposplenia, and non‐hematologic malignancies. While other laboratory findings were inconsistent with the presence of an active infection, the absence of prior malignancy with treatment also reduced the likelihood of leukemia. However, preliminary pathology of the paraspinal lumbar mass conducted in the hospital confirmed the presence of a granulocytic sarcoma [7, 24, 25]. According to guidelines from the National Comprehensive Cancer Network (NCCN), multidisciplinary testing including immunohistochemistry, cytochemistry, and molecular genetic analysis is needed to diagnose AML or any of its subtypes in accordance with the 2016 World Health Organization (WHO) classification system [13, 14]. To meet diagnostic criteria for APL, APL morphology must be confirmed, along with the presence of either t(15:17) and/or PML‐RARA.

The rarity of extramedullary leukemia without a prior history of AML or any of its subtypes requires additional confirmatory testing. After results from the bone marrow biopsy of the L3 vertebrae showed an absence of leukemic blast cells, further consultation with a third‐party hematopathologist was necessary to confirm the presence of AML or any of its subtypes and rule out other pathological causes of the paraspinal mass. At first, immunophenotypic studies discovered several genes associated with AML, including MPO, CD33, lysozyme, and CD68, suggesting that the paraspinal mass was a myeloid neoplasm. However, a FISH analysis of the remaining biopsy samples was recommended to confirm whether the mass was an extramedullary form of APL. The subsequent FISH analysis discovered the presence of PML‐RARA and t(15;17), confirming the diagnosis of extramedullary APL.

Treatment for nearly each reported case of extramedullary APL includes a combination of 7 + 3 induction chemotherapy, followed by a course of ATRA [3, 13]. 7 + 3 induction chemotherapy consists of an anthracycline, usually either daunorubicin or idarubicin, administered for 3 days at the beginning of treatment given concurrently with cytarabine for 7 days. Venetoclax was added to the patient's course given high risk of relapse [9]. One recent variation on the treatment of primary presenting extramedullary APL includes the addition or substitution of ATRA with ATO [8, 18, 23, 26]. With two exceptions, the majority of cases treated with 7 + 3 induction chemotherapy followed by ATRA, regardless of variation, experienced partial or complete remission after treatment. Of the two exceptions, one was unresponsive to chemotherapy and expired due to rapidly progressive intratumoral hemorrhage, while the other experienced relapse 1 year after initial treatment, with subsequent ATO and two rounds of chemotherapy resulting in remission [23, 27]. Another notable treatment option in a previous case of primary extramedullary APL included surgical resection of the paraspinal mass, though the composition of the mass was unknown at the time of procedure [28]. For the present study, a neurosurgery consultation obtained early during hospitalization recommended against resection of the paraspinal mass. In general, a review of the current literature revealed that treatment for primary extramedullary APL consisting of chemotherapy followed by ATRA possessed the most benefit.

### 5.2 Case Discussion

This case highlights a rare primary presentation of extramedullary APL in the form of a myeloid sarcoma (chloroma) of the lumbar spine, leading to CES. While APL is typically diagnosed through peripheral blood and bone marrow findings, this patient lacked bone marrow involvement at presentation, making the diagnosis more challenging.

The differential diagnosis initially included lymphoma, metastatic disease, and primary spinal tumors, given the presence of a lumbar paraspinal mass on imaging. The detection of metamyelocytes in laboratory findings raised suspicion for leukemia; however, a normal bone marrow biopsy from the L3 vertebra complicated the diagnostic process [3, 13, 28]. Ultimately, immunophenotypic analysis and FISH studies of the paraspinal mass confirmed the presence of PML‐RARA fusion and t(15;17), establishing a diagnosis of extramedullary APL [13, 24, 29]. This case underscores the importance of recognizing extramedullary leukemia as an initial presentation of APL, even in the absence of overt bone marrow involvement. Clinicians should maintain a high index of suspicion in patients presenting with neurologic deficits, spinal masses, and laboratory findings suggestive of hematologic malignancy, as prompt diagnosis and initiation of chemotherapy are critical to improving patient outcomes.

Extramedullary APL is an uncommon variant of APL in which leukemic blasts infiltrate tissues outside the bone marrow. This condition is particularly rare in the absence of prior hematologic malignancy. In this case, the patient's large paraspinal myeloid sarcoma exerted a mass effect on the cauda equina, resulting in neurologic symptoms characteristic of CES.

Importantly, chloromas can be the first sign of underlying leukemia, preceding bone marrow involvement [9]. Therefore, early identification and initiation of chemotherapy, even in the absence of bone marrow findings, can be lifesaving. The observed tumor regression following induction chemotherapy further supported its leukemic origin. Standard induction therapy for APL, which includes ATRA and ATO or anthracycline‐based regimens, was initiated in this patient [8, 18, 23, 26]. The rapid resolution of symptoms and tumor shrinkage reinforced the importance of early intervention in extramedullary APL.

While extramedullary APL is associated with a variable prognosis, early diagnosis and appropriate therapy can significantly improve outcomes. Long‐term monitoring is necessary to assess for bone marrow relapse, which remains a concern in cases with primary extramedullary involvement.

## Author Contributions

**Amir Ali:** investigation, validation. **Marc Ringor:** conceptualization, data curation, investigation, writing – original draft. **Arbab Khalid:** conceptualization, data curation, formal analysis, writing – original draft. **Diana Garcia:** data curation, investigation, writing – original draft. **Ravishankar Konchada:** investigation, writing – original draft. **Ramalingam Ratnasabapathy:** investigation, methodology, supervision. **Yuna Chae:** investigation, writing – review and editing. **Arman Melikyan:** investigation, writing – review and editing. **George Yaghmour:** project administration, validation.

## Funding

The authors have nothing to report.

## Ethics Statement

The authors have nothing to report.

## Consent

The authors have nothing to report.

## Conflicts of Interest

The authors declare no conflicts of interest.

## Data Availability

All datasets and software used for supporting the conclusions of this case study are found on patient records at Keck Medicine of USC Cerner.
